# Intra‐Sectoral Differences in Climate Change Strategies: Evidence from the Global Automotive Industry

**DOI:** 10.1002/bse.1968

**Published:** 2017-06-06

**Authors:** Matthias Damert, Rupert J. Baumgartner

**Affiliations:** ^1^ Institute for Systems Sciences, Innovation and Sustainability Research (ISIS) and FWF‐DK Climate Change University of Graz Graz Austria

**Keywords:** climate change, corporate strategy, automotive industry, supply chain, institutional environment, content analysis

## Abstract

Companies are increasingly challenged for action on climate change. Most studies on business responses to climate change focus on cross‐sector comparisons and neglect intra‐sectoral dynamics. This paper investigates the influence of supply chain position and regional affiliation on climate change strategies within a particular industry. We present a generic framework integrating both market and non‐market responses to climate change. We argue that climate change strategies comprise several corporate activities that have different foci of interaction and four main objectives: governance, innovation, compensation and legitimation. Using a global sample of 116 automotive companies, we conduct a cluster analysis and identify four types of strategy. We find that the sophistication of automobile manufacturers' strategies significantly differs from that of suppliers. Regional affiliation and firm size prove to be determinants of the strategy type pursued. We cannot find evidence for a relationship between financial performance and a company's strategic approach to climate change. © 2017 The Authors. Business Strategy and the Environment published by ERP Environment and John Wiley & Sons Ltd

## Introduction

In recent years, increasing pressure has been exercised on businesses to consider climate change as a relevant strategic issue. Before the ratification of the Kyoto Protocol in 2005, companies focused on socio‐political action, i.e. non‐market responses, e.g. through influencing law‐making procedures to be favorable for their business (Kolk and Pinkse, 2005; Lee, 2012a). However, the number and stringency of climate regulations increased in subsequent years, accompanied by a rising environmental awareness of the public and investor requests for transparency on corporate greenhouse gas (GHG) emissions and strategies to reduce them. This resulted in a shift towards market responses, i.e. proactive managerial and technological measures*,* such as the establishment of carbon inventories, investments in ‘green’ products and cleaner production processes (Jeswani *et al.*, 2008; Weinhofer and Hoffmann, 2010).

Businesses in the transportation sector are particularly targeted by demands for climate change action, as the sector accounts for 22% of global carbon dioxide (CO_2_) emissions (IPCC, 2014). GHG emissions from this sector are growing at a faster rate than in any other energy end‐use industry. Passenger and freight transport alone is responsible for about three‐quarters of total sector emissions (IEA, 2013). Against this background, various policies such as fuel economy standards and emission‐based taxes on cars aim to reduce CO_2_ emissions from vehicle use, including the CAFE regulations in the US, vehicle taxes in the EU or similar approaches in other major markets such as China and Japan (ICCT, 2014). To achieve emission reduction targets requires the introduction of new technologies (e.g. alternative propulsion systems), new services (e.g. fleet management systems) and different usage patterns of automobiles (e.g. car sharing) (IPCC, 2014). While original equipment manufacturers (OEMs) are mainly held responsible for vehicle emissions, suppliers increasingly play a key role in efforts to cut emissions. Due to increased outsourcing, a large portion of innovation is nowadays done by suppliers (Böttcher and Müller, 2015). In the case of the German automotive industry, for example, suppliers generate about 70% of value added (Di Botonto, 2014). The success of OEMs' responses to climate change is therefore heavily dependent on the performance of their suppliers (Lee, 2011).

In management science, the analysis of corporate action on climate change is a comparatively recent development. Although numerous scholars have examined companies' general approaches to sustainability and environmental protection, few studies opted to specifically analyze the characteristics of climate change strategies (e.g. Kolk and Pinkse, 2005; Weinhofer and Hoffmann, 2010; Lee, 2012a) and their determinants (e.g. Okereke, 2007; Boiral *et al.*, 2012; Böttcher and Müller, 2015). Moreover, previous research exhibits three main limitations. First, categorizations of strategies primarily rely on cross‐sector analyses (e.g. Jeswani *et al.*, 2008; Sprengel and Busch, 2011; Backman *et al.*, 2017). Little research has studied the variation of strategies within sectors (Weinhofer and Hoffmann, 2010). Such an investigation can yield valuable knowledge about intra‐industrial dynamics and can control for external influences that are otherwise difficult to grasp due to sector‐specific institutional environments. Intra‐sectoral analyses are especially suited to better understand the importance of the so‐called ‘home‐country effect’ on strategies (Levy and Kolk, 2002), suggesting that corporate action is shaped by the institutional setting of a company's main location. Second, the supply chain context of corporate climate action has largely been neglected so far (Jira and Toffel, 2013). To our knowledge, there is no research that looks at differences in strategies between companies in the same supply chain. Since the success of focal companies' responses to climate change is often closely related to the performance of their suppliers, analyzing the variation in strategies can yield valuable insights into the role of the supply chain position for action on climate change (Lee, 2011). Third, studies on the automotive industry in particular are rare, and focus on companies from a single country or case studies of only a few companies (e.g. Böttcher and Müller, 2015; Busch and Schwarzkopf, 2013). Moreover, a comparison between strategies of suppliers with those of OEMs has not been made so far. Consequently, there is a lack of an ample sector analysis that considers strategic responses of both OEMs and suppliers in different institutional environments.

Motivated by these research gaps, the research objective of the present paper is to develop a better understanding of the characteristics and determinants of corporate climate change strategies in the automotive industry, whilst taking into account supply chain and institutional aspects. Our contribution to the literature is threefold. First, we develop a generic framework for studying corporate responses to climate change from a holistic perspective by including both market and non‐market responses. Second, we provide empirical evidence for the influence of companies' home countries' institutional environment on climate change strategies. Third, our study contributes to the limited knowledge about differences in corporate action on climate change between companies in the same supply chain.

The remainder of the paper is structured as follows. First, we conduct a review of the literature on business responses to climate change and develop a research framework. We argue that climate change strategies consist of a set of different activities with four major strategic objectives: governance, innovation, compensation and legitimation. With the help of a content and a subsequent cluster analysis, we apply our framework to the global automotive industry. Based on a sample of 116 companies, we identify different types of strategy and investigate the influence of supply chain position, regional affiliation, firm size and financial performance.

## Literature Review and Research Framework

In the following, we provide a definition for corporate climate change strategy and depict activities that can be part of it. We then show how these measures relate to different strategic objectives and derive a research framework. Finally, we present factors influencing the implementation of strategies.

### Corporate Climate Change Strategy

In the literature, various terms have been used for companies' responses to climate change. When looking at the different approaches, it becomes apparent that many scholars exclusively focus on the management of corporate CO_2_ emissions (e.g. Jeswani *et al.*, 2008; Weinhofer and Hoffmann, 2010; Cadez and Czerny, 2016). Although corporate carbon emissions do indeed play a central role in the debate about climate change mitigation, solely concentrating on emission reduction neglects other important facets of business strategies, such as political lobbying, stakeholder management and corporate citizenship. In order to draw a more complete picture, we therefore consider both market and non‐market aspects of strategies in our study. By building on notions from Okereke and Russel (2010) and Busch and Schwarzkopf (2013), we define a corporate climate change strategy as a set of market and non‐market activities that aim to reduce and legitimize the impact of a firm's business activities on climate change. The scope of our research is hence limited to climate change mitigation. Corporate adaptation to the impacts of climate change is not considered.

### Research Framework for Corporate Climate Change Strategies

Previous studies have identified several types of activity that firms implement in response to climate change. By building on this research, we propose a comprehensive set of 11 categories of activities: GHG management and policy development, organizational involvement, risk management, product improvements, process improvements, new market and products, supplier involvement, emission trading and compensation, sector and stakeholder cooperation, corporate reporting, and political activities. These activities comprise several possible measures and practices that are depicted in Table 1.

**Table 1:** Corporate activities in response to climate change

Corporate activity	Specific measures and practices
GHG management and policy development	Emission inventory and implementation of environmental management systems (Gallego‐Alvarez, 2012; Jeswani *et al.*, 2008) Emission reduction targets (Lee, 2012a) Tracking and benchmarking emissions (Kolk and Pinkse, 2005; Jeswani *et al.*, 2008) Consideration of climate change aspects in investments (e.g. disinvesting from carbon‐intensive business segments) (Dunn, 2002)
Organizational involvement	Assigning climate change responsibilities to managers or committees (Boiral, 2006) Raising awareness and promoting behavioral change (e.g. energy‐efficiency workshops, substitution of business trips with video conferences) (Okereke, 2007) Monetary (e.g. remuneration based on corporate emission performance) and non‐monetary incentives (e.g. energy‐efficiency awards) (Backman *et al.*, 2017)
Risk management	Assessing challenges and opportunities regarding regulation and consumer perceptions (Sullivan, 2010) Integration of climate change into risk management and design of risk mitigation strategies (Weinhofer and Busch, 2013)
Product improvements	Assessing product emissions through lifecycle analyses (Lee, 2012a) Product innovation policy (Yunus *et al.*, 2016) Decrease share of carbon‐intensive products (Sprengel and Busch, 2011) Substitution of carbon‐intensive inputs (Böttcher and Müller, 2015)
Process improvements	Assessing emissions of production processes (Böttcher and Müller, 2015) Energy‐efficient equipment or plant retrofit (Zhang *et al.*, 2012) Improving logistic operations and switching to sustainable energy sources (Haddock‐Fraser and Tourelle, 2010)
New markets and products	Partnerships with other companies, governments and research institutions to develop products or enter markets in which low‐carbon aspects are important (Kolk and Pinkse, 2005; Jeswani *et al.*, 2008; Lee, 2012b)
Supplier involvement	Requesting suppliers to implement environmental management systems and to report emissions (Sullivan, 2010) Setting emission reduction targets in cooperation with suppliers (Lee, 2012a) Assisting suppliers in implementing measures (Lee, 2012b)
Emission trading and compensation	Acquiring emission allowances or compensating emissions through ETS or offsetting projects (Kolk and Pinkse, 2005; Weinhofer and Hoffmann, 2010)
Sector and stakeholder cooperation	Collaboration with other companies, political actors or NGOs (Kolk and Pinkse, 2007a) Voluntary initiatives with local communities (Jeswani *et al.*, 2008) Stakeholder dialogues (Sprengel and Busch, 2011)
Corporate reporting	Disclosure of climate change related information (Gallego‐Alvarez, 2012; Jeswani *et al.*, 2008)
Political activities	Lobbying, information campaigns, funding of research or political parties (Kolk and Pinkse, 2007a) Voluntary commitments as self‐regulation (Eberlein and Matten, 2009)

Based on the literature on climate change strategies, we argue that the above‐mentioned activities can be grouped along four main strategic intents: *governance*, *innovation*, *compensation* and *legitimation*. *Governance* refers to an organization's capabilities of dealing with risks and opportunities related to climate change mitigation and resulting governance mechanisms (Boiral, 2006; Tang and Luo, 2014) and can be broken down into three corporate activities: GHG management and policy development, organizational involvement, and risk management. *Innovation* embraces activities that are geared towards the technological improvement of existing products and processes and the development of new ones with the aim of reducing GHG emissions and gaining competitive advantages (Kolk and Pinkse, 2005). *Compensation* ‘describes the action taken by a company to balance or offset its CO_2_ emissions, such as buying CO_2_ credits or enhancing carbon sinks’ (Weinhofer and Hoffmann, 2010) while leaving ‘a company's own technological assets and competencies merely unaltered’ (Kolk and Pinkse, 2005). This includes the reduction of a company's indirect emissions through supplier involvement. The fourth objective, *legitimation*, summarizes activities aimed at retaining or gaining legitimacy for doing the respective business. While some of these measures can be of a rather symbolic nature, others relate to compliance issues, political influence and transparency (Boiral, 2006; Talbot and Boiral, 2015).

**Figure 1:**
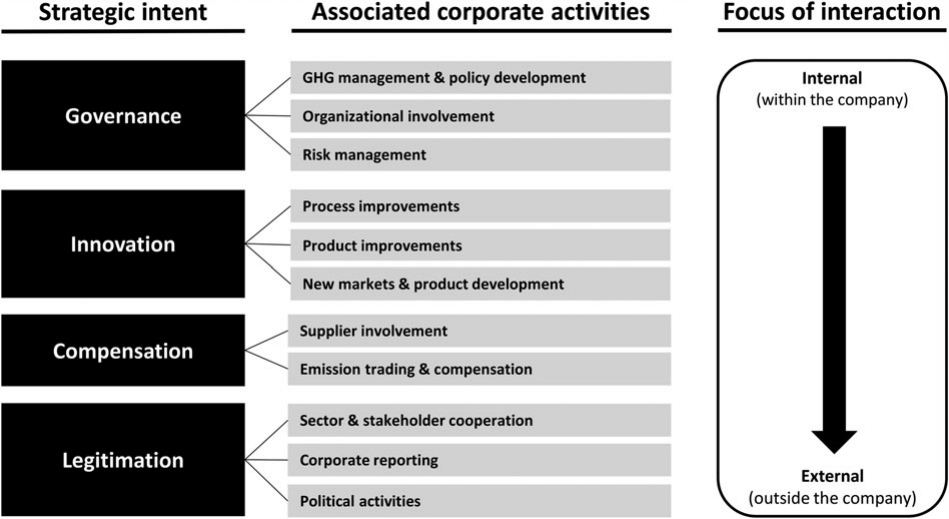
Corporate climate change strategy framework

Apart from distinct strategic intents, we argue that corporate activities in response to climate change have two foci of interaction: (1) an internal focus, i.e. through collaboration within the company, and (2) an external focus, i.e. through collaboration with actors outside of the company (Kolk and Pinkse, 2005). External activities can be carried out in cooperation with actors along the supply chain (vertical interaction), with companies in the same sector that are not part of the supply chain and with companies from other sectors (horizontal interaction) or with actors in the socio‐political environment including politicians, research institutes, the public and NGOs (contextual interaction). The 11 corporate activities, their corresponding strategic intent and their focus of interaction are summarized in a corporate climate change strategy framework shown in Figure 1.

The literature suggests that conceptualizations of climate change strategies can employ either a continuum‐ or a typology‐based perspective (Kolk and Mauser, 2002; Lee, 2012a). Continuum approaches view strategies as an evolutionary process, in which a firm incrementally transitions from a resistant to a proactive attitude towards climate change mitigation. Therefore, a continuum perspective stipulates that there is an ideal strategy, namely the most proactive one. Typology‐based approaches on the other hand do not emphasize a hierarchy or the temporal development of strategies but rather on different types of coexisting strategic approach. This notion implies that there is no one strategic optimum but rather a multitude of distinct strategies to reach the same goal.

The strategy framework proposed in the present study (Figure 1) can be considered to integrate both continuum‐ and typology‐based viewpoints and is hence similar to previous classifications in the research field (see, e.g., Kolk and Pinkse, 2005; Weinhofer and Hoffmann, 2010; Lee, 2012a). The framework employs a (static) typology perspective because it assumes that companies' strategies can exhibit different patterns. On the one hand, companies may implement none or any possible combination of the above‐mentioned climate change activities. On the other hand, firms may also differ in their prioritization of strategic intents, e.g. one company focuses on legitimizing its carbon footprint while another primarily aims at reducing GHG emissions. The proposed climate change strategy framework furthermore exhibits continuum characteristics because it assumes that firms differ with regard to the implementation level of activities. It therefore captures the dynamic aspects of corporate strategies by accounting for a possible evolution of climate change responses over time.

### Determinants of Corporate Climate Change Strategies

#### Institutional Environment

Institutional theory suggests that firms' action is shaped by three main kinds of institutional pressure: (1) normative, i.e. social and cultural norms to which companies are expected to adhere; (2) coercive, i.e. regulations and laws; (3) mimetic, i.e. the imitation of competitors' behavior to avoid uncertainty (DiMaggio and Powell, 1983). It also implies that in a particular organizational field strategic approaches will resemble each other in the long run due to similar institutional pressures, which is called institutional isomorphism (DiMaggio and Powell, 1983). Most research in the context of climate change provides evidence for the influence of different institutional environments on corporate responses (e.g. Cadez and Czerny, 2016; Tang and Luo, 2014). Most of these studies draw conclusions based on cross‐sector analyses. So far, few studies have focused on intra‐sectoral strategy dynamics. Levy and Kolk (2002) and Weinhofer and Hoffmann (2010) show that, in the case of multinational oil and electric utility companies, the institutional framework of a company's home country does indeed have an influence on corporate strategies. On the other hand, a case study of car manufacturers from different countries by Busch and Schwarzkopf (2013) finds little variation in strategies among the examined companies. This would support the idea of institutional isomorphism. It also questions the importance of the so‐called ‘home‐country effect’ (Levy and Kolk, 2002). If highly globalized companies with different regional affiliations exhibit similar strategy patterns, the institutional environments of a company's dominant sales markets might be more influential than home‐country factors, which is called the ‘host‐country effect’ (Amran *et al.*, 2016). While the goal of this paper is not to test hypotheses on the relationship between institutional factors and strategies, we want to shed more light on how the regional context of a company's business affects the type of strategy employed.

#### Supply Chain Position

In the literature on sustainable supply chain management, a popular topic is the relationship between internal and external factors and the adoption of environmental practices among suppliers (Zhu *et al.* 2007; Long and Young, 2016). Interestingly, there are few empirical investigations of whether climate change strategies of suppliers indeed differ from those pursued by their focal companies (Jira and Toffel, 2013). According to Kolk and Pinkse (2007b, p. 374), ‘the position of the company in the supply chain determines to which extent a company depends on its customers and thus follows a concomitant climate strategy’. While suppliers predominantly conduct business‐to‐business activities, OEMs are closer to the end consumer and therefore susceptible to a higher degree of stakeholder pressure (Bansal and Roth, 2000; Haddock‐Fraser and Tourelle, 2010). On this basis, we expect that, compared with suppliers, automobile manufacturers generally pursue more proactive strategies in response to climate change.

#### Firm Size and Financial Performance

Both the size of a company and its access to financial resources have often been adduced to explain variation in climate change strategies (Engau and Hoffmann, 2009; Weinhofer and Hoffmann, 2010; Lee, 2012a). The larger and economically more successful a firm is, the more sophisticated its strategy tends to be. Size and financial resources seem to go hand in hand, as larger firms usually have easier access to financial capital and possess more human resources for implementation purposes. Moreover, larger companies are also exposed to increased stakeholder scrutiny due to their higher visibility and media attraction (Bansal, 2005). We therefore expect firms that are comparatively large and perform better in financial terms to show a higher implementation level of activities in response to climate change.

## Research Design

### Data Collection and Sample

For two reasons, we deem the automotive industry particularly suitable for our study. First, as shown in the introduction, automotive companies play a crucial role in decarbonizing the transportation sector and are hence exposed to a variety of climate policies. Second, since the sector is also characterized by an extensive and global supply chain network, it represents a promising object of investigation for disentangling the variation of strategies across both countries and companies along the supply chain.

The sampling procedure followed a four‐step approach. In a first step, we constructed a database of the 550 largest automotive companies worldwide. We combined information on annual sales from different databases from industry groups, including the top 50 OEMs ranking of the Organisation Internationale des Constructeurs d'Automobiles (OICA), the top 500 automotive suppliers ranking from www.marklines.com, and technical journals, such as *Automobil Produktion* and *Automotive News*.

Second, we assessed suppliers' shares of automotive sales based on corporate financial data. Various automotive suppliers also sell their products to non‐automotive markets. We assumed that corporate strategies are mainly determined by the dominant business of a company. Consequently, we did not consider suppliers with automotive sales accounting for less than 50% of total sales.

In a third step, we collected publicly available company documents, including annual and sustainability reports, corporate websites, CDP reports, codes of conduct, purchasing guidelines, mission statements and press releases. In this regard, CDP (formerly known as the Carbon Disclosure Project) served as the main source. The CDP questionnaire has been used by several other studies in the field and has proved to be a valuable and comprehensive source of information (Weinhofer and Hoffmann, 2010; Jira and Toffel, 2013; Backman *et al.*, 2017). To increase the reliability of data, only companies with at least two different types of available document (e.g. CDP and annual report or annual and sustainability report) remained in the final sample. Due to region‐specific accounting and publishing policies, dates used for delimitating fiscal years varied across company reports. Therefore, we considered documents covering both the years 2013 and 2014, while we took only the most recent publications into account. Eventually, we had compiled 430 documents from 116 companies that fulfilled the sampling requirements.

After determining the final sample, we screened databases from national and international automotive trade associations and business initiatives (e.g. WBCSD and UN Global Compact) to see whether companies were members of an organization that is involved in lobbying activities. We also extracted information from company reports and financial websites about the location of companies' headquarters, firm size and financial performance. To better capture the influence of the institutional environment on corporate strategies, we decided to group the companies according to their home country's quality of climate mitigation policies measured by an index developed by the European Bank for Reconstruction and Development (EBRD, 2011). Based on the location of their headquarters, we assigned firms to the following regions (mentioned in order of the extensiveness of policies): European Union, Japan and South Korea, USA and Canada, and others (including India, Pakistan, South Africa and Turkey). Table 2 shows the composition of the final sample in terms of position in the supply chain and regional affiliation.

**Table 2:** Supply chain position and regional affiliation of companies in the sample

Location of headquarters	OEMs	Suppliers	Total
	35 (30.2%)	81 (69.8%)	116 (100.0%)
Japan and South Korea	12	39	51 (44.0%)
EU	14	25	39 (33.6%)
USA and Canada	4	11	15 (12.9%)
Others	5	6	11 (9.5%)

### Data Analysis

The data analysis was carried out in four steps. First, we analyzed the company documents through a qualitative content analysis with the software MAXQDA. We derived the category system for the content analysis from the strategy framework (see Figure 1). The 11 corporate activities were converted into categories for the coding procedure. Following recommendations from Neuendorf (2002) and based on a review of scientific articles and the obtained company documents, we developed a codebook containing detailed descriptions, examples and synonymic terms for the categories. In order to check whether the codebook is comprehensive and correctly reflects the categories, we discussed it with two other researchers who are experts in business responses to climate change. After this, the company documents were coded. During the content analysis, the category system proved to be exhaustive. Thus, no amendments had to be made.

In Step 2, we assessed the implementation level of each corporate activity. For this purpose, we developed a rating scheme that comprised the same categories as the content analysis. The underlying question for the rating scale was ‘What is the implementation level of the respective corporate activity?’. A five‐point Likert scale served to assign ‘scores’ to each company for each category. The scale ranged from 0 (very low implementation level) to 4 (very high implementation level). To accurately capture the extent to which activities can be theoretically implemented, we iteratively adapted the rating scheme to the context of each corporate activity. This was done through a preliminary screening of the coded documents and by comparing the rating scheme with similar ones used in previous studies (e.g. Kolk and Pinkse, 2005; Lee, 2012b; Weinhofer and Hoffmann, 2010). The final rating schemes can be found in the appendix. Finally, two researchers independently assessed the implementation level of activities. Inter‐rater reliability amounted to 87.9% (percentage of agreement) and 0.72 (Krippendorff's alpha), which is acceptable (Krippendorff, 2013). For distinct ratings, we made a consensus‐based decision on the respective final score to be used. We then assigned a score to the four strategic objectives (governance, innovation, compensation and legitimation) by computing the mean rating of the associated corporate activities.

To identify different types of strategy, the third step comprised a two‐step cluster analysis conducted in SPSS. Using the scores of the four strategic intents as input variables, we conducted a hierarchical cluster analysis to identify the optimal number of clusters. We chose the Ward method and squared Euclidean distance as the distance measure. After this, we plotted the proximity measures of each stage of the clustering procedure in a scree plot and applied the elbow criterion. It turned out that four clusters were the optimal solution. Subsequently, a non‐hierarchical cluster analysis (*k*‐means) was used to assign the companies to the four strategy clusters (Hair *et al.*, 2010).

In the last step, we performed a Mann–Whitney *U* test and an ANOVA to statistically analyze the differences between OEMs and suppliers on the one hand and the different strategy clusters on the other hand. We applied the non‐parametric Mann–Whitney *U* test to compare the implementation levels of corporate activities, because a Shapiro–Wilk test showed a non‐normal distribution of the data. Due to multiple comparisons between OEMs and suppliers we adjusted the significance level through a Šidák correction to avoid making a ‘Type 1’ error, i.e. rejecting the null hypothesis when it is true (Abdi, 2007). A one‐way ANOVA served to control differences in firm size and financial performance among the identified strategy clusters. Firm size was measured by the natural logarithm of annual sales while financial performance was measured by return on assets (ROA) and return on equity (ROE). This is in line with similar studies (e.g. Sprengel and Busch, 2011; Lee, 2012a).

## Results and Discussion

### Descriptive Statistics

Table 3 shows the characteristics of the analyzed firms. On average, automobile manufacturers are larger than suppliers in terms of both annual sales and workforce. Profitability is relatively high and little variation exists in this regard. When looking at the level of corporate action (Table 4), it becomes apparent that automotive companies are generally highly active in implementing measures in response to climate change. Most of the companies have realized the risks and opportunities related to climate change, set up GHG inventories, adopted emission reduction targets and show a high degree of organizational involvement. Moreover, the majority of businesses in the sector have introduced ambitious product and process innovations that already led to emission reductions and explore new markets in which low‐carbon aspects are a unique selling point.

**Table 3:** Average firm size and financial performance of analyzed companies

	OEMs	Suppliers	Total
Firm size
Total annual sales [million USD]	55 331	10 623	24 112
No of employees	96 791	46 086	61 518
Financial performance
ROA [%]	5.3	5.5	5.4
ROE [%]	16.0	13.5	14.3

**Table 4:** Implementation level of activities and supply chain position

Strategic intent and associated corporate activity	Total	OEMs	Suppliers	Mann–Whitney *U* test
	Mean (SD)	Mean (SD)	Mean (SD)	*p* value
Governance
GHG management and policy development	3.5 (0.796)	3.7 (0.622)	3.4 (0.851)	0.088
Organizational involvement	3.1 (0.737)	3.5 (0.562)	3.0 (0.758)	0.001+
Risk management	2.7 (1.633)	3.2 (1.431)	2.5 (1.674)	0.010
Innovation
Product improvements	3.6 (0.759)	3.7 (0.631)	3.5 (0.808)	0.356
Process improvements	3.8 (0.651)	4.0 (0.169)	3.7 (0.755)	0.023
New markets and products	3.1 (1.570)	3.7 (0.950)	2.8 (1.696)	0.001+
Compensation
Supplier engagement	2.2 (1.334)	3.1 (1.105)	1.9 (1.246)	0.000+
Emission trading and compensation	0.8 (1.432)	1.8 (1.734)	0.4 (1.060)	0.000+
Legitimation
Sector and stakeholder cooperation	2.8 (1.331)	3.5 (0.507)	2.5 (1.459)	0.000+
Corporate reporting	2.4 (1.227)	2.9 (1.255)	2.1 (1.152)	0.001+
Political activities	2.4 (0.856)	2.9 (0.974)	2.2 (0.729)	0.000+

*α* = 0.00465 after Šidák correction (initially *α* = 0.05);

+ *p* < 0.004 65, otherwise not significant

Most companies exhibit a low to medium level of measures that externally reduce emissions, such as supplier engagement and emission offsetting. The acquisition of additional emission credits in particular is a barely pursued measure. Thus, the results indicate that a large share of companies prefer reducing emissions through technological measures over compensating them (Table 4). Firms also actively engage with their stakeholders and peers through corporate citizenship activities and voluntary business initiatives, respectively. On the other hand, political lobbying and carbon disclosure seem to be of less importance for the analyzed companies since they are implemented to a lower degree. Moreover, the data shows that activities related to governance and innovation are preferred to activities aimed at legitimizing a company's business operations. This is in line with previous research that revealed a transition from non‐market to market responses in recent years (Kolk and Pinkse, 2005; Lee, 2012a).

The descriptive statistics also indicate significant differences between automotive suppliers and OEMs. On average, manufacturers show a higher level of implementation in every category (Table 4). The Mann–Whitney *U* test shows that OEMs' strategies are in particular more advanced than those of suppliers with regard to organizational involvement (*p* < 0.001), exploring new markets (*p* < 0.001), compensating GHG emissions (*p* < 0.000), disclosing climate change related information (*p* < 0.001) and seeking cooperation with external actors, including suppliers (*p* < 0.000), other companies and stakeholders (*p* < 0.000) and policy‐makers (*p* < 0.000). The lowest variance exists in terms of emission reduction policies, risk management procedures and technological measures, i.e. product and process improvements, underlined by non‐significant *p* values.

### The Climate Change Strategy Clusters

The cluster analysis yielded four distinct groups of corporate strategies. The pseudo‐*F* value calculated during the *k*‐means clustering procedure confirmed that the four clusters are a meaningful classification of the data set. Table 5 provides a summary of the cluster centers, i.e. the mean score of respective strategic actions, and the number of companies in each cluster. It is noteworthy that companies are almost evenly distributed across the clusters. Firms also exhibit similar strategy patterns, i.e. innovation is of highest and compensation of lowest priority, and there is a hierarchy of clusters in terms of proactivity, illustrated by the average strategy score of the clusters. Hence, there is a continuum ranging from strategies that are still in the planning phase (‘introverted laggard’) to those that are very ambitious (‘all‐round enhancer’). This supports other studies on environmental strategies and corporate responses to climate change that outlined an evolution of corporate action on a continuum (e.g. Kolk and Mauser, 2002; Jeswani *et al.*, 2008). The different clusters are explained in the following.

**Table 5:** The four climate change strategy clusters and mean scores

Strategic intent	Cluster			
	All‐round enhancer	Legitimating reducer	Emergent innovator	Introverted laggard
Governance	3.83	3.73	2.98	2.08
Innovation	3.91	3.75	3.78	2.42
Compensation	3.42	1.37	1.17	0.48
Legitimation	3.52	3.02	2.19	1.60
Ave. score	3.67	2.97	2.53	1.65
Number of cases	25 (21.6%)	27 (23.3%)	36 (31.0%)	28 (24.1%)

#### All‐Round Enhancer

Companies in this cluster exhibit a very high implementation level in all four categories of climate change management activities. They represent about one‐fifth of the whole sample and can be characterized as the most proactive companies in the sector. All‐round enhancers resemble similar strategy types found in other business sectors, e.g. ‘all‐round explorer’ (Lee, 2012a), ‘all‐rounder’ (Weinhofer and Hoffmann, 2010) or ‘active’ (Jeswani *et al.*, 2008).

#### Legitimating Reducer

This strategy cluster consists of companies that are very active both in building up governance capabilities for climate change issues and in innovating for carbon emission reductions. They also actively engage with their stakeholders and regulatory bodies in order to gain legitimacy for their actions. At the same time, however, initiatives related to emission credit acquisition and reducing suppliers' emissions are still in the planning phase. Respective companies thus mainly focus on emission reduction activities and on legitimizing their business operations rather than on compensating their emissions externally. Legitimating reducers represent approximately one‐fourth of the automotive companies analyzed and are similar to the strategy types ‘substituting reducer’ (Weinhofer and Hoffmann, 2010) and ‘emission avoiders’ (Sprengel and Busch, 2011) identified in previous research.

#### Emergent Innovator

The top priority of corporate strategies in this cluster is to innovate for achieving carbon emission reductions. The corresponding companies exhibit the second‐highest score in this regard. While ambitious innovation policies have been implemented and are comparable to those of the first two clusters, governance capabilities are less developed. Similarly to legitimating reducers, emergent innovators only show a low level of compensation activities. Moreover, basic external cooperation and communication processes are in place. These companies represent about one‐third of the whole sample.

#### Introverted Laggard

The fourth cluster can be characterized as the least active one. Although firms of this group display a similar strategy pattern to emergent innovators, they clearly lag behind their competitors and score lowest in all categories. While respective companies are generally aware about environmental issues, governance capabilities are still at an early stage. The same goes for innovation policies. Firms are aware of their carbon footprint but have not yet implemented measures to reduce emissions from their products and production processes. The possibility of compensating emissions is not considered in most cases. Moreover, stakeholder and political engagement is on a low level. In conclusion, such companies concentrate on internal activities. This strategy type is comparable to categorizations in other studies, e.g. ‘indifferent’ companies (Jeswani *et al.*, 2008) or ‘wait‐and‐see observers’ (Lee, 2012a).

### Differences Between the Strategy Clusters

Table 6 illustrates how companies are spread over the clusters according to their position in the supply chain and their regional affiliation (represented by their headquarters' location). Table 7 shows how firm size (measured by the natural logarithm of annual sales in million USD and the number of employees, respectively) and financial performance (expressed by ROA and ROE) vary across the clusters.

**Table 6:** Distribution of companies across the strategy clusters

	Cluster			
	All‐round enhancer	Legitimating reducer	Emergent innovator	Introverted laggard
Supply chain position
OEMs	51.4%	22.9%	17.1%	8.6%
Suppliers	8.6%	23.5%	37.0%	30.9%
Regional affiliation
Japan and South Korea	15.7%	25.5%	43.1%	15.7%
EU	30.9%	25.6%	17.9%	25.6%
USA and Canada	26.7%	20.0%	33.3%	20.0%
Others	9.1%	18.2%	9.1%	63.6%
All companies (116)	21.6%	23.3%	31.0%	24.1%

**Table 7:** Differences regarding firm size and financial performance

	Cluster				ANOVA *F* value
	All‐round enhancer	Legitimating reducer	Emergent innovator	Introverted laggard	
Firm size
Total annual sales [log]	10.7	9.2	8.9	8.0	28.19*
No of employees [log]	11.5	10.2	10.1	9.5	14.95*
Financial performance
ROA [%]	4.4	4.6	5.4	7.2	1.41
ROE [%]	20.2	9.9	15.1	12.3	2.26
No of companies	25 (21.6%)	27 (23.3%)	36 (31.0%)	28 (24.1%)	

* *p* < 0.001, otherwise not significant

In line with the descriptive statistics (see Table 4), it can be concluded that OEMs are generally more proactive than suppliers (Table 6). 73.3% of the former belong to the two most active clusters, while the latter are mostly in the two clusters that have the least ambitious strategies (67.9%). More than half of the OEMs pursue an ‘all‐round enhancer’ type of strategy, whereas companies further up the supply chain are predominantly ‘emergent innovators’. An explanation could be that being closer to the end consumer translates into more sophisticated action due to greater visibility and exposure to public scrutiny (Bansal and Roth, 2000; Haddock‐Fraser and Tourelle, 2010). Due to their focus on business‐to‐business activities, suppliers are likely to be the least visible actors in the automotive industry. Consequently, the degree of stakeholder pressure is much lower and so is the need for ambitious action on climate change.

The fact that European automobile manufacturers are mainly ‘all‐round enhancers’ would also explain why compared with the other clusters this strategy type's focus is much more on emission compensation. Companies with large manufacturing facilities in the EU are subject to higher regulatory pressure, as they are obliged to participate in the European ETS when a certain emission threshold is crossed. This applies to most of the European‐based OEMs and is underlined by the fact that on average ‘all‐round enhancers’ are the largest firms in the sample (Table 7).

Differences also exist regarding the regional affiliation of a company (Table 6). The relative majority of European firms are ‘all‐round enhancers’ (30.9%), whereas North American firms are mostly ‘emergent innovators’ (33.3%). However, firms from these regions are relatively evenly distributed across the clusters. Thus, their strategies seem to be influenced not solely by their home‐country's institutional environment but rather by other factors, such as the framework conditions of other countries they are operating in (Amran *et al.*, 2016). Companies that are more globalized are exposed to a greater variety of institutional settings and tend to show more ambitious action on climate change to legitimate their business (Bansal, 2005).

In the case of companies from Japan, South Korea and ‘other’ countries, the results are less ambiguous. Almost half of the Japanese and South Korean businesses are emergent innovators and hence mainly focus on emission reductions through innovation. Apart from this, there is a tendency that these firms are on the less active side of the strategy spectrum (Table 6). This is an indication of a more internal focus of the strategies pursued. A reason could be that the institutional environment in Japan and South Korea incentivizes technological change, while putting less emphasis on corporate reporting practices and stakeholder engagement. Moreover, the climate policy regimes in Japan and South Korea are less stringent compared with e.g. the EU, inter alia because of the absence of an ETS and less ambitious national emission reduction targets (EBRD, 2011). This might also explain why Japanese and South Korean firms are predominantly in clusters with a lower implementation level of compensation, governance and stakeholder‐related measures.

The majority of automotive companies from other countries are part of the ‘introverted laggard’ cluster (63.6%). This can be attributed to a lower average regulatory pressure that companies from these countries are exposed to. Climate policies in these countries are relatively weak, and therefore the reduction and legitimation of corporate carbon footprints might not be the highest priority for the respective companies (EBRD, 2011).

The empirical analysis confirms that the size of a company affects the type of strategy employed (Table 7). The ANOVA results show that the average firm size of the clusters differs significantly, in terms of both annual sales (*p* < 0.001) and number of employees (*p* < 0.001). The greater the average firm size of a cluster, the more proactive is the respective group of companies. These findings support two main lines of argumentation in the literature. First, larger companies demonstrate more sophisticated action due to their greater visibility and exposure to a higher degree of institutional pressure. Second, larger companies also tend to possess more organizational resources to implement measures in response to climate change (Engau and Hoffmann, 2009; Weinhofer and Hoffmann, 2010; Lee, 2012a).

In contrast to the significant influence of firm size on strategies, the relationship between financial performance and corporate climate action is inconclusive (Table 7). Based on the ANOVA, we had to reject the hypotheses that the different strategy clusters significantly differ from each other in this regard. Hence, the economic performance of a company seems not to be linked to the type of climate change strategy pursued, supporting findings of previous research in the field (Lee, 2012a).

## Conclusion

This study illustrates how the institutional environment of a company's home country and its position in the supply chain affect action on climate change. The empirical analysis shows that international automotive companies are generally very active in implementing climate change measures. However, strategies of OEMs and suppliers differ significantly from each other. Apart from technological solutions to emission reductions, OEMs are more ambitious in almost every aspect, confirming that closeness‐to‐consumer and associated stakeholder pressure can play a crucial role in shaping business responses to climate change (Sprengel and Busch, 2011). This is supported by a cluster analysis. While strategies in the automotive industry exhibit similar patterns in terms of the prioritization of objectives, they can be mapped on a continuum from less to more active. Automobile manufacturers mainly belong to clusters with a high overall level of climate change action, whereas suppliers are mostly located at the other end of the spectrum. Moreover, OEMs focus much more on carbon compensation measures and engagement with stakeholders and policy‐makers to legitimate their business operations.

The comparison of climate change strategy types with regard to different institutional environments yielded ambiguous results. European and North American businesses tend to be most proactive. However, there seems to be no dominant type of strategy pursued, because companies from both regions are relatively evenly distributed across the strategy clusters. Companies from Japan and South Korea, although exposed to more stringent climate policy regimes than their North American counterparts, exhibit a lower level of strategy implementation. Nevertheless, their strategic priorities are more distinct, expressed in a preference for technological solutions to emission reductions over legitimizing or compensating them. Climate actions of companies from the above‐mentioned regions are thus likely determined by factors other than their home country's institutional environment. In contrast to this, companies from countries with the least ambitious climate regulations also tend to be in the least active strategy clusters, which would in turn emphasize the importance of regulatory pressure for proactive climate action.

We also illustrated that firm size affects the type of strategy pursued. Larger firms implement more sophisticated measures in response to climate change, probably due to their greater exposure to public scrutiny and easier access to financial and human resources. Yet, we could not find an empirical relationship between a company's financial situation and the type of strategy it pursues, which is in line with previous studies (Lee, 2012a).

Our study has important implications for businesses and policy‐makers. First, the findings support other researchers' call for a more thorough integration of suppliers in emission reduction efforts (Böttcher and Müller, 2015). Due to a lower level of stakeholder pressure further upstream in the supply chain, focal companies should make use of their buyer power to stimulate more ambitious action on climate change on the part of their suppliers. Economic rationales might be another reason for suppliers' resistance to be more proactive. However, other researchers have shown that the ambitiousness of strategies can be associated with financial benefits (e.g. Boiral *et al.*, 2012), and our analysis at least does not hint at a negative relationship between financial performance and climate action. Second, since suppliers are generally smaller in size and possess less organizational resources compared with OEMs, policies that incentivize corporate contributions to climate change mitigation, such as subsidies or tax incentives for renewable energy use, should especially target smaller firms.

There are five avenues that are particularly promising for future research. First, we could not fully explain the strategic responses to climate change of European and North American businesses. A more detailed analysis of institutional characteristics could shed light on the effectiveness of specific policies in triggering corporate action. Second, there remains a need for investigations of strategy dynamics within an industry, as the results of the present study might not be applicable to other business sectors. While the nature of an intra‐sector study reduces the generalizability of findings, it helps to isolate the effects of sector‐specific regulatory and stakeholder pressures. Third, case studies of highly globalized companies could generate more insights into the mechanism of action of the home‐country and host‐country effects. Studying companies that operate in a variety of markets could help to disentangle whether the level of influence of the home country's policy regime is connected to the degree of a company's internationalization. Fourth, the literature still lacks longitudinal studies of corporate climate action, its determinants and effectiveness in generating long‐term outcomes. Last, little research has addressed business responses to climate adaptation issues. As the impacts of climate change are likely to be felt soon, understanding the drivers and types of corporate adaptation strategies is of vital importance for policy‐makers to design appropriate regulation.

## Rating Schemes for Corporate Activities

### GHG Management and Policy Development

**Table:**

Rating	The company…
0	does not have/mention GHG inventory or reduction targets
1	has intentions/plans for estimating emissions
2	has estimated emissions but has no reduction targets
3	has estimated its emissions and wants to reduce its emissions, without providing details
4	has estimated its emissions, has reduction targets, and tracks emissions

### Organizational Involvement

**Table:**

Rating	The company…
0	does not mention/has no plans for organizational involvement
1	has intentions/plans for organizational involvement
2	has implemented *one* of the following measures: activities to raise employees' awareness related to climate change assigned climate change responsibilities to top‐level managers/committees incentives for achieving emission reduction targets (e.g. variable remuneration)
3	has implemented *two* of the above‐mentioned measures
4	has implemented *all* the above‐mentioned measures

### Risk Management

**Table:**

Rating	The company…
0	does not mention or has no plans for risk management
1	has intentions/plans for assessing risk and opportunities related to climate change
2	views itself not exposed to climate change risks or opportunities, without providing details
3	mentions general risks/opportunities (e.g. market trends or legislation) related to climate change, but climate change not integrated into risk management procedures
4	has integrated climate change into risk management procedures and provides details

### Product Improvements

**Table:**

Rating	The company…
0	does not mention or has no plans for product improvements
1	has intentions/plans for product improvements, without providing any details
2	has estimated product emissions (e.g. through LCA), but does not provide examples of product improvements
3	provides examples of product improvements, but does not provide details about related emission reductions
4	provides examples of product improvements and provides details about related emission reductions (e.g. weight reduction, fuel consumption)

### Process Improvements

**Table:**

Rating	The company…
0	does not mention or has no plans for process improvements
1	has intentions or plans for process improvements, without providing details
2	has estimated process emissions (e.g. related to energy consumption), but does not provide examples of process improvements
3	provides examples of process improvements (e.g. new energy‐saving equipment), but does not provide details about emission reductions
4	provides examples of process improvements and provides details related to emission reductions (e.g. improvements in energy efficiency, carbon emissions saved)

### New Markets and Products

**Table:**

Rating	The company…
0	does not mention or does not plan to launch products or enter markets related to low‐carbon aspects
1	has intentions or plans for launching products or entering markets, but does not provide details
2	has intentions or plans for launching products or entering markets and provides details
3	prepares launch of products or market entrance (e.g. research partnerships, pilot projects, prototypes, acquisition of a company)
4	has launched products or entered new markets

### Supplier Engagement

**Table:**

Rating	The company…
0	does not mention supplier engagement or has no plans to engage them
1	has intentions/plans for engaging suppliers
2	has a general supply chain policy (e.g. green procurement guidelines, code of conduct) related to environmental protection without specific relation to climate change
3	requests its suppliers to implement environmental management systems or to reduce emissions
4	requests its suppliers to implement environmental management systems or to reduce emissions, and assists in implementing emission reduction measures

### Emission Trading and Compensation

**Table:**

Rating	The company…
0	does not mention or has not purchased emission credits via ETS or compensation/offsetting schemes
1	has intentions/plans for acquiring emission credits or for compensation/offsetting, without providing details
2	is preparing for ETS or voluntary compensation/offsetting initiatives (e.g. pilot project or working group for ETS design)
3	has acquired emission credits *or* is taking part in voluntary emission compensation/offsetting initiatives (e.g. CDM, JI)
4	has acquired emission credits *and* is taking part in voluntary emission compensation/offsetting initiatives

### Sector and Stakeholder Cooperation

**Table:**

Rating	The company…
0	does not mention or does not plan to engage in stakeholder initiatives related to climate change
1	has intentions/plans for engaging in stakeholder initiatives, but does not provide details
2	has intentions/plans for engaging in stakeholder initiatives and provides details
3	engages in *one* of the following activities: stakeholder dialogues/roundtables on climate change issues (e.g. for creating materiality matrix) corporate citizenship initiatives related to climate change mitigation (e.g. tree planting, public education events, research funding)
4	engages in *both* above‐mentioned activities

### Corporate Reporting

**Table:**

Rating	The company…
0	reports about climate change aspects but does not have any formal reporting policy on environmental or climate‐related disclosure
1	reports about climate change aspects and has a formal reporting policy, but does not refer to any reporting guidelines
2	reports about climate change aspects and refers to specific reporting guidelines (e.g. CDP, GRI, ISO26000), but has no rating
3	publicly reports about climate change aspects according to GRI, CDP or similar standards and has a rating
4	reports about environmental issues according to GRI, CDP or similar standards and ranked/scored among the highest scoring band (CDP score above 90, GRI A+)

### Political Activities

**Table:**

Rating	The company…
0	does not engage in political activities or does not mention it
1	is stating its opinion on climate change policies without mentioning its engagement in political activities
2	engages in *one* of the following political activities related to climate change: working together with trade associations or business initiatives funding of research organizations direct lobbying of policy‐makers
3	engages in *two* of the above‐mentioned political activities
4	engages in *all* above‐mentioned political activities
